# Adjusting Reported COVID-19 Deaths for the Prevailing Routine Death Surveillance in India

**DOI:** 10.3389/fpubh.2021.641991

**Published:** 2021-08-05

**Authors:** Hemant Deepak Shewade, Giridara Gopal Parameswaran, Archisman Mazumder, Mohak Gupta

**Affiliations:** ^1^International Union Against Tuberculosis and Lung Disease (The Union), Paris, France; ^2^The Union South-East Asia Office, New Delhi, India; ^3^All India Institute of Medical Sciences, New Delhi, India

**Keywords:** coronavirus, mortality, cause of death ascertainment, death registration, missing deaths

## Abstract

In India, the “low mortality” narrative based on the reported COVID-19 deaths may be causing more harm than benefit. The extent to which COVID-19 deaths get reported depends on the coverage of routine death surveillance [death registration along with medical certification of cause of death (MCCD)] and the errors in MCCD. In India, the coverage of routine death surveillance is 18.1%. This is compounded by the fact that COVID-19 death reporting is focused among reported cases and the case detection ratio is low. To adjust for the coverage of routine death surveillance and errors in MCCD, we calculated a correction (multiplication) factor at national and state level to produce an estimated number of COVID-19 deaths. As on July 31, 2020, we calculated the infection fatality ratio (IFR) for India (0.58:100–1.16:100) using these estimated COVID-19 deaths; this is comparable with the IFR range in countries with near perfect routine death surveillance. We recommend the release of excess deaths data during COVID-19 (at least in states with high death registration) and post-mortem COVID-19 testing as a surveillance activity for a better understanding of under-reporting. In its absence, we should adjust reported COVID-19 deaths for the coverage of routine death surveillance and errors in MCCD. This way we will have a clear idea of the true burden of deaths and our public health response will never be inadequate. We recommend that “reported” or “estimated” is added before the COVID-19 death data and related indicators for better clarity and interpretation.

## Background

On March 11, 2020, COVID-19 (a respiratory illness caused by novel coronavirus—SARS-CoV-2) was declared as a pandemic by the World Health Organization (WHO) (1, 2). Globally, as on July 31, 2020, around 675 000 people had succumbed to this disease (3). In India, the first case was reported on January 30, 2020. On July 31, 2020, the country had the third highest burden globally in terms of reported cases (1.12 million) (3). A total of 12 003 tests per million (TPM) were conducted with a test positivity rate of 6.8%. There were 35 747 reported COVID-19 deaths which translated to 26 reported COVID-19 deaths per million (DPM) (4). As per WHO and Indian Council of Medical Research (ICMR), if it is medically certified that the underlying cause of death is confirmed or suspected COVID-19, then it should be recorded and reported as a COVID-19 death (5, 6).

The case fatality ratio (CFR) measures fatality by dividing the reported COVID-19 deaths by reported COVID-19 cases. The cases are detected through surveillance (and are highly under-reported) and this crude method of calculation of mortality gives rise to variable estimates of CFR by country—from <0.1:100 to over 25:100 (7). The infection fatality ratio (IFR) is a better measure of fatality as the denominator includes total estimated infections. Both CFR and IFR can be low if under-reporting of COVID-19 deaths is high and/or only confirmed COVID-19 deaths are reported. Globally, many countries have stopped focusing on CFR as it is not a good predictor of overall mortality from SARS-CoV-2 and is not recommended for evaluation of policy or comparison across settings (7–9).

In September 2020, India was in the news for the highest number of reported COVID-19 cases per day and reached the second spot globally in terms of the cumulative reported cases (3). However, the reported COVID-19 DPM and reported CFR were low (3). The under-reporting of COVID-19 deaths could be one of the potential reasons. An editorial published in *The Lancet* (September 26, 2020) highlighted the issue of transparency of data on COVID-19 deaths in India and warned against the dangers of ensuing false optimism due to under-reporting (10).

Hence, we discuss the routine deaths surveillance in India, the rationale for adjusting the reported COVID-19 deaths for coverage of the routine death surveillance and its implications on the estimated IFR for India.

## Routine Death Surveillance in India

The civil registration system report (latest being CRS 2018, when this perspective piece was written) provides the national and state-wise coverage of death registration by dividing the registered deaths with estimated deaths (11). The national and state-wise coverage of medical certification of cause of death (MCCD) among registered deaths is provided by the MCCD report (latest being 2018, when this perspective piece was written) (12). Estimates of deaths are provided by the sample registration system where continuous enumeration of births and deaths is done in a sample of villages/urban blocks by a resident part time enumerator. This is verified by an independent six monthly retrospective survey by a full time supervisor (13).

In India, the coverage of death registration is 86% and coverage of MCCD among registered deaths in 21% (11, 12). Populous states like Uttar Pradesh, Bihar, and Jharkhand that constitute 30% of the population of India and have very low coverage of death registration (35–61%) (11). Other states with low death registration are Telangana (58%), Assam (66%), Arunachal Pradesh (48%), Manipur (28%), and Nagaland (10%) (11). Many states have 100% death registration but MCCD coverage lower than the national average (Odisha, Kerala, Rajasthan, Punjab) (11, 12).

Routine death surveillance includes death registration along with MCCD (11, 12). We multiplied the coverage of death registration among estimated deaths (source: CRS 2018) with the coverage of MCCD among registered deaths (source: MCCD 2018) to obtain the coverage of routine death surveillance (11, 12). At the national level, this was 18.1%. Therefore, the prevailing coverage of routine death surveillance in India is poor. The reason being only 34% received institutional medical attention at the time of death and not all hospitals have been brought under the coverage of MCCD (11, 12).

The state-wise coverage of routine death surveillance is depicted in Table 1. It was <10% in Jharkhand, Nagaland, Uttar Pradesh, Bihar, Uttarakhand, and Madhya Pradesh; 60–75% in Delhi, Chandigarh, and Puducherry; and 100% in Goa.

**Table 1:** The State-wise prevailing coverage of routine death surveillance (death registration along with MCCD) and the correction factor, India (2018) (11, 12).

**S. No**.	**States**	**Death registration among estimated deaths**	**MCCD among registered deaths**	**Death registration along with MCCD**	**Correction (multiplication) factor to adjust for routine death surveillance**	**Range of combined correction (multiplication) factor to adjust for routine death surveillance and errors in MCCD**
		**A**	**B**	C = A^*^B	**D = 1/(C)**	Lower range = D^*^1, Upper range = D^*^2
1.	Andhra Pradesh	1.000	0.149	0.149	6.7	6.7, 13.4
2.	Telangana	0.582	0.374	0.218	4.6	4.6, 9.2
3.	Arunachal Pradesh	0.478	0.329	0.157	6.4	6.4, 12.8
4.	Assam	0.669	0.120	0.080	12.5	12.5, 25.0
5.	Bihar	0.346	0.136	0.047	21.3	21.3, 42.6
6.	Chhattisgarh	0.835	0.198	0.165	6.1	6.1, 12.2
7.	Goa	1.000	1.000	1.000	1.0	1.0, 2.0
8.	Gujarat	1.000	0.234	0.234	4.3	4.3, 8.6
9.	Haryana	1.000	0.204	0.204	4.9	4.9, 9.8
10.	Himachal Pradesh	0.839	0.150	0.126	8.0	8.0, 16.0
11.	Jharkhand	0.549	0.046	0.025	39.6	39.6, 79.2
12.	Karnataka	1.000	0.311	0.311	3.2	3.2, 6.4
13.	Kerala	1.000	0.119	0.119	8.4	8.4, 16.8
14.	Madhya Pradesh	0.788	0.105	0.083	12.1	12.1, 24.2
15.	Maharashtra	0.984	0.348	0.342	2.9	2.9, 5.8
16.	Manipur	0.375	0.514	0.193	5.2	5.2, 10.4
17.	Meghalaya	0.897	0.431	0.387	2.6	2.6, 5.2
18.	Mizoram	1.000	0.589	0.589	1.7	1.7, 3.4
19.	Nagaland	0.097	0.287	0.028	35.9	35.9, 71.8
20.	Odisha	1.000	0.111	0.111	9.0	9.0, 18.0
21.	Punjab	1.000	0.171	0.171	5.9	5.9, 11.8
22.	Rajasthan	0.999	0.131	0.131	7.6	7.6, 15.2
23.	Sikkim	1.000	0.425	0.425	2.4	2.4, 4.8
24.	Tamil Nadu	1.000	0.450	0.450	2.2	2.2, 4.4
25.	Tripura	1.000	0.223	0.223	4.5	4.5, 9.0
26.	Uttarakhand	0.707	0.111	0.078	12.7	12.7, 25.4
27.	Uttar Pradesh	0.608	0.051	0.031	32.3	32.3, 64.6
28.	West Bengal	0.918	0.129	0.118	8.4	8.4, 16.8
29.	Andaman and Nicobar	0.729	0.595	0.434	2.3	2.3, 4.6
30.	Chandigarh	1.000	0.718	0.718	1.4	1.4, 2.8
31.	Delhi	1.000	0.623	0.623	1.6	1.6, 3.2
32.	Puducherry	1.000	0.740	0.740	1.4	1.4, 2.8
33.	J&K and Ladakh	0.633	–	0.633	1.6	1.6, 3.2
34.	DNH, D&D	0.857	1.000	0.857	1.2	1.2, 2.4
35.	**India**	**0.860**	**0.210**	**0.181**	**5.5**	**5.5, 11**

*MCCD, medical certification of cause of death; J and K, Jammu and Kashmir; DNH, Dadra Nagar Haveli; D and D, Daman and Diu*.

* *The correction factor to adjust for the prevailing routine death surveillance may be multiplied with a correction factor for errors in MCCD (we have considered an upper limit of two) to get the combined correction factor to adjust for the prevailing routine death surveillance as well as errors in MCCD. The combined correction factor could be higher than the upper range if errors in MCCD are higher than our assumptions*.

Among deaths undergoing registration along with MCCD, errors during MCCD are also common in developing countries like India (14–18). Therefore, the quality of MCCD is also poor.

## Adjusting for Routine Death Surveillance and Errors in MCCD

### Rationale

**Figure 1:**
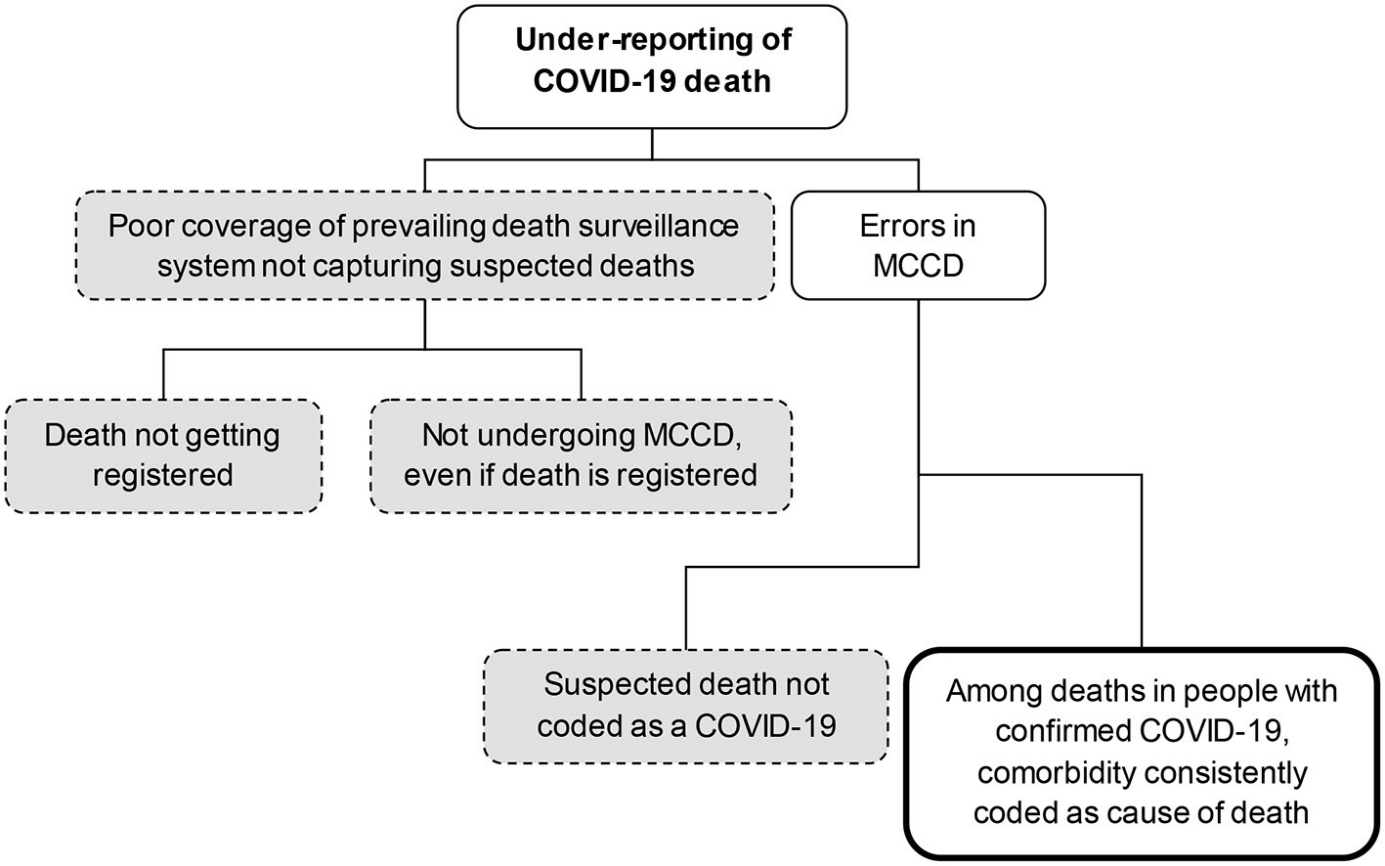
The levels/causes of under-reporting of COVID-19 deaths*. MCCD, medical certification of cause of death. *Dotted and shaded boxes related to the under-reporting of COVID-19 deaths among non-reported cases, Bold and non-shaded box related to under-reporting among confirmed cases.

Only a minority of all COVID-19 cases are getting recorded through testing and are reported. The second national seroprevalence survey in August–September 2020 suggested that the case detection ratio was around 6–7:100 (19). For the purpose of this exercise, as on July 31, 2020, it was assumed that the case detection ratio was at least 5:100. The extent to which COVID-19 deaths get reported among the large subset (95% or above) of unreported COVID-19 in the community depends on (i) the prevailing coverage of routine death surveillance; and (ii) extent of errors in MCCD (Figure 1). Within the reported COVID-19 cases also, the COVID-19 deaths could be missed due to the poor coverage of routine death surveillance and errors in MCCD. Despite WHO and ICMR guidelines (5, 6), the suspected COVID-19 deaths are not getting reported in India (20). The COVID-19 illness also presents a myriad of clinical presentations post-recovery that may lead to mortality which may not be captured by the present system in India (21).

Therefore, before we compare and interpret the differences in the reported COVID-19 deaths and the corresponding indicators of India with countries having near 100% routine death surveillance (Supplementary Table 1), we should adjust for the prevailing coverage and quality of routine death surveillance. This also applies to interpretation of differences across states within India.

### Correction Factor for the Coverage of Routine Death Surveillance

At the national and the state level, the correction (multiplication) factor was inverse of the coverage of routine death surveillance. This factor varied from state to state. It was 5.5 nationally, (100/18.1) and ranged from 40 (100/2.5) for Jharkhand, to none (100/100) for Goa (Table 1). We calculated the estimated COVID-19 deaths, nationally and state wise, by multiplying the correction factor with the reported COVID-19 deaths. As on July 31, 2020, the estimated COVID-19 deaths in India were 196 997; an estimated 144 DPM (Supplementary Table 2).

As on July 31, 2020, Uttar Pradesh, Madhya Pradesh, and Jharkhand were not in the top 10 states based on reported DPM. When we adjusted for routine death surveillance and calculated the estimated DPM (Supplementary Table 2), they were in the top 10. The ranks of Bihar (25 to 17) and West Bengal (10 to 6) jumped upward (Supplementary Figure 1). Incidentally, these were the only five states that had <10 000 TPM. These five states constitute 43% of the national population. Based on reported COVID-19 deaths, these five states constituted 12% of total COVID-19 deaths but based on estimated COVID-19 deaths, they constituted 43% of the total COVID-19 deaths. The correlation coefficient (r) between TPM and coverage of routine death surveillance was 0.673 (*p* < 0.001).

As we used routine death surveillance data from 2018 and applied it to the year 2020, we also did sensitivity analysis for the correction factor by assuming different percentage increases in coverage of routine death surveillance between 2018 and 2020 (Supplementary Table 3). However, we do not expect any significant improvement as at the national level over the past 9 years, death registration has only increased from 67 to 86%, while the MCCD among registered deaths has been stagnant at approximately 20% (11, 12).

### Correction Factor for Errors in MCCD

To adjust for under-reporting due to errors in MCCD among the deaths captured by routine death surveillance, we did not have an objective state-wise and national estimate of the error. There were state- and city-specific modeling exercises and media reports where the reported COVID-19 deaths (due to errors in MCCD) were as low as 50% (correction factor of two) of the actual deaths reported from hospitals (22, 23). We assumed two correction factors for errors in MCCD (1.5 and 2). We arrived at the estimated COVID-19 deaths after adjusting for errors in MCCD (Supplementary Table 2). The errors in MCCD explain why for a state like Goa with 100% routine death surveillance, under-reporting of COVID-19 deaths is still possible.

### Combined Correction Factor

By multiplying the correction factor for error in MCCD and the correction factor for the prevailing routine death surveillance, we calculated a combined correction factor for the poor coverage and the quality of the routine death surveillance (Table 1). At the national level, the overall under-reporting could therefore possibly be by a factor as low as 5.5 (5.5^*^1) or as high as 11 (5.5^*^2) (Table 1). It could be higher than 11 if the upper limit for correction factor for errors in MCCD is more than two. Using a correction factor of 11, as on July 31, 2020, the estimated deaths could be as high as 393 932 (288 DPM) (Supplementary Table 2). Similarly, we calculated the range of combined correction factor for states (Table 1).

### The Estimated COVID-19 Infection Fatality Ratio in India

We created a matrix to calculate the estimated IFR for India as on July 31, 2020 taking a range of scenarios for the estimated COVID-19 deaths and the case detection ratio (Supplementary Table 4). With a correction factor ranging from 5.5–11 and case detection ratio of 5:100, the estimated IFR ranged from 0.58:100–1.16:100.

## Discussion

### Key Message

We arrived at an estimate of the COVID-19 under-reporting in India using publicly available indicators regarding the coverage of routine death surveillance and some assumptions in errors in MCCD. Hence, there are two levels of under-reporting. First, COVID-19 death not being captured by routine death surveillance. Second, though the death is captured, the COVID-19 death is missed due to error in MCCD (Figure 1). This is also relevant for other countries with poor routine death surveillance (countries in south-east Asia and Africa). A strong positive correlation between TPM and the prevailing coverage of routine death surveillance suggests that states with weak mortality surveillance were also the ones who had conducted fewer tests.

To put the estimated COVID-19 deaths in perspective (around 200 000–400 000 as on July 31, 2020), every year there are an estimated 127 000 (95% CI: 64 046, 190 139) influenza- associated deaths in India. With respect to the under-reporting of deaths, an estimated 450 000 TB- related deaths occur annually in India which is 5.6 times the reported TB deaths (24–26).

### Cannot Compare Apples With Oranges

As on July 31, 2020, of the top 10 countries (based on reported cases), eight had 90%−100% coverage of routine death surveillance (India: 18%, Peru: 57%) and all had higher reported COVID-19 DPM than India (3, 27). Small countries like the UK and Belgium with high DPM tested almost 25% of their population; have a robust routine death surveillance system and they reconcile COVID-19 deaths from their routine death surveillance system (3, 27). Other countries like Brazil, Mexico, Chile, and Peru that have a similar age distribution and socioeconomic status also have higher reported DPM when compared to India (28). The difference could be explained by their better routine death surveillance (Supplementary Table 1). As on May 13, 2021, when we compared with countries in south-east Asia, where most of the countries have a poor routine death surveillance (27), India had the highest reported COVID-19 DPM (3). Additional factors to consider before comparing DPM are the estimated cases based on seroprevalence surveys, death definitions used, testing strategies adopted, response to the epidemic, prevalence of comorbidities, quality of medical care, and population density.

Countries with high coverage of routine death surveillance may also miss COVID-19 deaths due to errors during MCCD or if they do not reconcile deaths from routine death surveillance. But the overall extent of under-reporting is expected to be lower when compared to countries with poor death surveillance as chances of missing deaths (irrespective of the cause) and cause-specific deaths are comparatively less due to robust mortality surveillance systems.

### The COVID-19 Mortality Rate in India Is Similar to Global Figures

Using reported deaths in the numerator, the ICMR study (at around 1:100 case detection ratio in May–June) suggested the IFR to be 0.15:100 in high stratum districts and 0.01:100 in low stratum districts (29). The IFR estimated by us for India (0.58:100–1.16:100) is within the range estimated in countries with near perfect routine death surveillance (0.5:100 in Switzerland to 1.4:100 in Italy) and similar to the point estimate (0.68:100) based on a systematic review and meta-analysis (30, 31).

### Other Methods to Calculate the Estimated COVID-19 Deaths

The combined correction factor provides a bird's-eye view. The best way to estimate the COVID-19 under-reporting using publicly available data would be to factor in excess deaths during the COVID-19 epidemic as compared to previous years with a sub-group analysis of age groups and cause of death (if available). The difference between excess deaths and reported COVID-19 deaths will give an idea about the possible extent of under-reporting (32). Subject to availability of granular data, exact number of excess deaths attributed to COVID-19 may be calculated by adjusting for rate of change in the number of registered deaths over the past few years, decrease in accident- and pollution-related deaths during COVID-19, and a possible increase in deaths due to disruption in routine health services. Another option is to look for excess home deaths (33). In India, the place of death (home or health facility) is a variable collected during death registration (11). The “Excess deaths” analysis is a practical option for cities, districts and states with high coverage of death registration. However, with few exceptions, the excess deaths data has not been made public like elsewhere (34, 35).

In countries with high coverage of routine death surveillance, missed suspected deaths (due to error in MCCD) can also be identified based on an increase in deaths due to pneumonia, respiratory failure, sepsis or ill-defined causes higher than the maximum limit for the number of weekly occurrences of each cause (36).

In addition to excess deaths analysis, another feasible option is post-mortem surveillance. This would mean testing all registered deaths in a surveillance area for a specific surveillance period for COVID-19 at frequent intervals. This is feasible and not resource intensive. A study from Zambia (2020) suggested that of the 364 deaths around the tertiary health facility of capital city Lusaka, 70 were COVID positive (51 in community and 19 in facility) based on post- mortem polymerase chain reaction testing. Of 70, only six were tested pre-mortem for COVID giving an under-reporting by a factor of 10 (37). Zambia has not reported the routine death surveillance coverage data to WHO indicating its routine death surveillance is poor (27).

### Policy Implications

The coverage of routine death surveillance cannot be improved overnight but errors in MCCD can be prevented among the captured deaths. We can ensure that all suspected COVID-19 deaths are tested and we do not wrongly assign co-morbidity as the cause of death in confirmed COVID-19 patients.

In India, by using reported deaths to infer mortality, we are indirectly encouraging states with poor surveillance and discouraging states with relatively good surveillance. This also does not create an environment of data transparency. This false optimism of “low mortality” based on reported deaths could result in laxity among the administrators where appropriate mitigation measures may not be implemented. The health infrastructure may not be appropriately strengthened. The public health messaging based on this narrative may result in laxity among the public in following COVID19 appropriate behavior (10). The second wave that we witnessed in April–May 2021 in India could be a result of this “false optimism.” This could prove to be disastrous in states with poor routine death surveillance.

The administrators (local/state/national) should be encouraged to monitor and track the estimated COVID-19 deaths and act accordingly. If the administrators act only based on the reported deaths or only when the hospital infrastructure is over-stretched (only 34% deaths happen in hospitals in India) (11), it will be too little and too late.

## Limitations

There are some limitations. First, on July 31, the COVID-19 epidemic was relatively an urban phenomenon (with relatively better death surveillance) in India and our correction factor was at state level. Therefore, our correction factor could be an overestimate and it will be more representative of the true picture as the epidemic spreads from urban to rural areas (say late 2020 and 2021). On May 13, 2021, there were 258 317 reported COVID-19 deaths (reported 186 DPM) (4). This translates to an estimated 1023–2046 DPM if we apply the combined correction factor ranging from 5.5 to 11. Second, the CRS and MCCD reports only provide state-level estimates of coverage because the sample registration system (source for the denominator in the coverage indicators) is designed to provide state-level estimates (11–13). Finally, there is an inherent limitation in the death registration coverage indicator reported by CRS. The deaths registered in CRS (numerator) are based on the place of death while the estimated deaths from sample registration system (denominator) are based on place of usual residence (11, 13).

## Conclusion

The estimated COVID-19 deaths in India after adjusting for the coverage and quality of the routine death surveillance may be 5.5–11 times the reported COVID-19 deaths. The estimated COVID-19 deaths using IFR and DPM of India COVID-19 is comparable to the range in countries with robust death surveillance systems. Therefore, the COVID-19 fatality (rate and absolute numbers) in India does not appear to be lower than global figures.

The reported COVID-19 DPM and reported CFR/IFR of India cannot be compared with countries with robust death surveillance. We urge that “reported” or “estimated” be added before the COVID-19 death data and related indicators for better clarity and interpretation. The WHO should release country specific estimates for COVID-19 cases and deaths (like it does for other diseases); this will help in making meaningful and reliable comparisons.

In India, the epidemic is far from over and understanding the true picture of mortality will aid in eliciting an appropriate public health response and the masses adhering to the mitigation strategies (10). The “low mortality” narrative based on the reported COVID-19 deaths may be causing more harm than benefit. This should be treated as a unique window of opportunity to improve routine death surveillance.

## Data Availability Statement

The data is available on request from the corresponding author Hemant Deepak Shewade (hemantjipmer@gmail.com).

## Author Contributions

HS and GP conceived the idea and prepared the first draft. HS, GP, AM, and MG extracted the data. HS, GP, and AM analyzed, interpreted, and visualized the data. All authors critically reviewed the manuscript for important intellectual content and approved the final draft for submission.

## Conflict of Interest

The authors declare that the research was conducted in the absence of any commercial or financial relationships that could be construed as a potential conflict of interest.

## Publisher's Note

All claims expressed in this article are solely those of the authors and do not necessarily represent those of their affiliated organizations, or those of the publisher, the editors and the reviewers. Any product that may be evaluated in this article, or claim that may be made by its manufacturer, is not guaranteed or endorsed by the publisher.
