# Supplementary material for: Adjusting Reported COVID-19 Deaths for the Prevailing Routine Death Surveillance in India
Source: Front Public Health. 2021 Aug 5;9:641991. doi: 10.3389/fpubh.2021.641991 (PMC8374621; doi:10.3389/fpubh.2021.641991)
Supplement: Supplementary file 1 [file Data_Sheet_1.docx]

**Suppl Figure 1. Top ten states in India^@^ based on reported and estimated^ COVID-19 deaths per million in India, 31 July 2020***

**^@^**India reported deaths per million 26 and estimated deaths per million 144; ^after adjusting for the prevailing coverage of routine death surveillance (death registration along with medical certification of cause of death); *updated at 08 00 hours

**Suppl Table 1. Comparison^ of reported COVID-19 deaths per million and prevailing coverage of routine death surveillance in top ten countries (based on reported COVID-19 cases), 31 July 2020***[3]

| **Country** | **Reported COVID-19 deaths per million** | **Coverage (%) of routine death surveillance*** |
| --- | --- | --- |
| U.S.A | 477 | 100 |
| Brazil | 440 | 99 |
| India | 26 | 18 |
| Russia | 96 | 100 |
| South Africa | 137 | 92 |
| Mexico | 362 | 100 |
| Peru | 588 | 57 |
| Chile | 498 | 95 |
| Spain | 608 | 100 |
| Iran | 202 | 90 |

^Other factors to consider before comparing and inferring COVID-19 deaths per million are stage of the epidemic curve, COVID-19 death definitions used, the testing strategies adopted, response to the epidemic, prevalence of comorbidities, age distribution, quality of medical care and population density

*Deaths getting registered along with medical certification of cause of death

**Suppl Table 2. Estimated COVID-19 deaths after adjusting for prevailing coverage of routine death surveillance as well as adjusting for errors in MCCD, India, 31 July 2020***

| **State** | **Reported COVID-19 deaths** | **Correction factor for errors in MCCD** | **Estimated deaths after adjusting for errors in MCCD** | **Correction factor for prevailing death surveillance** | **Estimated deaths after adjusting for prevailing death surveillance** | **Correction factor for errors in MCCD and prevailing death surveillance** | **Estimated deaths after adjusting for errors in MCCD and prevailing death surveillance** |
| --- | --- | --- | --- | --- | --- | --- | --- |
|  | (A) | (B) | (C=A*B) | (D) | (E=A*D) | (F=B*D) | (G=C*D=A*F) |
| Andhra Pradesh | 1281 | 1 | 1281 | 6.7 | 8596 | 6.7 | 8596 |
|  | 1281 | 1.5 | 1922 |  |  | 10.1 | 12893 |
|  | 1281 | 2 | 2562 |  |  | 13.4 | 17191 |
| Arunachal Pradesh | 3 | 1 | 3 | 6.4 | 19 | 6.4 | 19 |
|  | 3 | 1.5 | 5 |  |  | 9.5 | 29 |
|  | 3 | 2 | 6 |  |  | 12.7 | 38 |
| Assam | 94 | 1 | 94 | 12.5 | 1171 | 12.5 | 1171 |
|  | 94 | 1.5 | 141 |  |  | 18.7 | 1757 |
|  | 94 | 2 | 188 |  |  | 24.9 | 2342 |
| Bihar | 282 | 1 | 282 | 21.3 | 5993 | 21.3 | 5993 |
|  | 282 | 1.5 | 423 |  |  | 31.9 | 8989 |
|  | 282 | 2 | 564 |  |  | 42.5 | 11985 |
| Chhattisgarh | 51 | 1 | 51 | 6.1 | 309 | 6.1 | 309 |
|  | 51 | 1.5 | 77 |  |  | 9.1 | 463 |
|  | 51 | 2 | 102 |  |  | 12.1 | 617 |
| Goa | 42 | 1 | 42 | 1.0 | 42 | 1.0 | 42 |
|  | 42 | 1.5 | 63 |  |  | 1.5 | 63 |
|  | 42 | 2 | 84 |  |  | 2.0 | 84 |
| Gujarat | 2418 | 1 | 2418 | 4.3 | 10325 | 4.3 | 10325 |
|  | 2418 | 1.5 | 3627 |  |  | 6.4 | 15487 |
|  | 2418 | 2 | 4836 |  |  | 8.5 | 20650 |
| Haryana | 417 | 1 | 417 | 4.9 | 2043 | 4.9 | 2043 |
|  | 417 | 1.5 | 626 |  |  | 7.4 | 3065 |
|  | 417 | 2 | 834 |  |  | 9.8 | 4087 |
| Himachal Pradesh | 14 | 1 | 14 | 8.0 | 111 | 8.0 | 111 |
|  | 14 | 1.5 | 21 |  |  | 11.9 | 167 |
|  | 14 | 2 | 28 |  |  | 15.9 | 223 |
| Jammu & Kashmir, | 372 | 1 | 372 | 1.6 | 588 | 1.6 | 588 |
| and Ladakh | 372 | 1.5 | 558 |  |  | 2.4 | 882 |
|  | 372 | 2 | 744 |  |  | 3.2 | 1176 |
| Jharkhand | 103 | 1 | 103 | 39.6 | 4079 | 39.6 | 4079 |
|  | 103 | 1.5 | 155 |  |  | 59.4 | 6118 |
|  | 103 | 2 | 206 |  |  | 79.2 | 8158 |
| Karnataka | 2230 | 1 | 2230 | 3.2 | 7181 | 3.2 | 7181 |
|  | 2230 | 1.5 | 3345 |  |  | 4.8 | 10771 |
|  | 2230 | 2 | 4460 |  |  | 6.4 | 14361 |
| Kerala | 70 | 1 | 70 | 8.4 | 588 | 8.4 | 588 |
|  | 70 | 1.5 | 105 |  |  | 12.6 | 882 |
|  | 70 | 2 | 140 |  |  | 16.8 | 1176 |
| Madhya Pradesh | 857 | 1 | 857 | 12.1 | 10361 | 12.1 | 10361 |
|  | 857 | 1.5 | 1286 |  |  | 18.1 | 15542 |
|  | 857 | 2 | 1714 |  |  | 24.2 | 20722 |
| Maharashtra | 14729 | 1 | 14729 | 2.9 | 43009 | 2.9 | 43009 |
|  | 14729 | 1.5 | 22094 |  |  | 4.4 | 64513 |
|  | 14729 | 2 | 29458 |  |  | 5.8 | 86017 |
| Manipur | 4 | 1 | 4 | 5.2 | 21 | 5.2 | 21 |
|  | 4 | 1.5 | 6 |  |  | 7.8 | 31 |
|  | 4 | 2 | 8 |  |  | 10.4 | 42 |
| Meghalaya | 5 | 1 | 5 | 2.6 | 13 | 2.6 | 13 |
|  | 5 | 1.5 | 8 |  |  | 3.9 | 19 |
|  | 5 | 2 | 10 |  |  | 5.2 | 26 |
| Mizoram | 0 | 1 | 0 | 1.7 | 0 | 1.7 | 0 |
|  | 0 | 1.5 | 0 |  |  | 2.6 | 0 |
|  | 0 | 2 | 0 |  |  | 3.4 | 0 |
| Nagaland | 5 | 1 | 5 | 35.9 | 180 | 35.9 | 180 |
|  | 5 | 1.5 | 8 |  |  | 53.9 | 269 |
|  | 5 | 2 | 10 |  |  | 71.8 | 359 |
| Odisha | 169 | 1 | 169 | 9.0 | 1523 | 9.0 | 1523 |
|  | 169 | 1.5 | 254 |  |  | 13.5 | 2282 |
|  | 169 | 2 | 338 |  |  | 18.0 | 3042 |
| Punjab | 370 | 1 | 370 | 5.9 | 2165 | 5.9 | 2165 |
|  | 370 | 1.5 | 555 |  |  | 8.8 | 3247 |
|  | 370 | 2 | 740 |  |  | 11.7 | 4329 |
| Rajasthan | 663 | 1 | 663 | 7.6 | 5065 | 7.6 | 5065 |
|  | 663 | 1.5 | 995 |  |  | 11.5 | 7598 |
|  | 663 | 2 | 1326 |  |  | 15.3 | 10131 |
| Sikkim | 1 | 1 | 1 | 2.4 | 2 | 2.4 | 2 |
|  | 1 | 1.5 | 2 |  |  | 3.5 | 4 |
|  | 1 | 2 | 2 |  |  | 4.7 | 5 |
| Tamil Nadu | 3838 | 1 | 3838 | 2.2 | 8520 | 2.2 | 8520 |
|  | 3838 | 1.5 | 5757 |  |  | 3.3 | 12781 |
|  | 3838 | 2 | 7676 |  |  | 4.4 | 17041 |
| Telangana | 505 | 1 | 505 | 4.6 | 2318 | 4.6 | 2318 |
|  | 505 | 1.5 | 758 |  |  | 6.9 | 3477 |
|  | 505 | 2 | 1010 |  |  | 9.2 | 4636 |
| Tripura | 21 | 1 | 21 | 4.5 | 94 | 4.5 | 94 |
|  | 21 | 1.5 | 32 |  |  | 6.7 | 141 |
|  | 21 | 2 | 42 |  |  | 9.0 | 188 |
| Uttarakhand | 76 | 1 | 76 | 12.7 | 968 | 12.7 | 968 |
|  | 76 | 1.5 | 114 |  |  | 19.1 | 1452 |
|  | 76 | 2 | 152 |  |  | 25.5 | 1936 |
| Uttar Pradesh | 1587 | 1 | 1587 | 32.3 | 51181 | 32.3 | 51181 |
|  | 1587 | 1.5 | 2381 |  |  | 48.4 | 76771 |
|  | 1587 | 2 | 3174 |  |  | 64.5 | 102362 |
| West Bengal | 1536 | 1 | 1536 | 8.4 | 12964 | 8.4 | 12964 |
|  | 1536 | 1.5 | 2304 |  |  | 12.7 | 19446 |
|  | 1536 | 2 | 3072 |  |  | 16.9 | 25928 |
| Andaman & Nicobar | 4 | 1 | 4 | 2.3 | 9 | 2.3 | 9 |
|  | 4 | 1.5 | 6 |  |  | 3.5 | 14 |
|  | 4 | 2 | 8 |  |  | 4.6 | 18 |
| Chandigarh | 14 | 1 | 14 | 1.4 | 19 | 1.4 | 19 |
|  | 14 | 1.5 | 21 |  |  | 2.1 | 29 |
|  | 14 | 2 | 28 |  |  | 2.8 | 39 |
| Dadra Nagar Haveli, | 2 | 1 | 2 | 1.2 | 2 | 1.2 | 2 |
| Daman & Diu | 2 | 1.5 | 3 |  |  | 1.8 | 4 |
|  | 2 | 2 | 4 |  |  | 2.3 | 5 |
| Delhi | 3936 | 1 | 3936 | 1.6 | 6337 | 1.6 | 6337 |
|  | 3936 | 1.5 | 5904 |  |  | 2.4 | 9505 |
|  | 3936 | 2 | 7872 |  |  | 3.2 | 12674 |
| Puducherry | 48 | 1 | 48 | 1.4 | 65 | 1.4 | 65 |
|  | 48 | 1.5 | 72 |  |  | 2.0 | 97 |
|  | 48 | 2 | 96 |  |  | 2.7 | 130 |
| **India** | 35747 | 1 | 35747 | 5.5 | 196966 | 5.5 | 196966 |
|  | 35747 | 1.5 | 53621 |  |  | 8.3 | 295449 |
|  | 35747 | 2 | 71494 |  |  | 11.0 | 393932 |

MCCD – medical certification of cause of death; Ladakh; *Updated at 08 00 hours, 31^st^ July 2020

**Suppl Table 3. Estimated^ COVID-19 deaths per million in India, 31 July 2020*: sensitivity analysis**

| Coverage of death registration | Coverage of MCCD among registered deaths | | | | | | | | |
| --- | --- | --- | --- | --- | --- | --- | --- | --- | --- |
|  | **0.211**** | **0.3** | **0.4** | **0.5** | **0.6** | **0.7** | **0.8** | **0.9** | **1.00** |
| 0.86** | 144 | 100 | 75 | 60 | 50 | 43 | 37 | 33 | 30 |
| 0.88 | 139 | 98 | 73 | 59 | 49 | 42 | 37 | 33 | 29 |
| 0.90 | 136 | 96 | 72 | 57 | 48 | 41 | 36 | 32 | 29 |
| 0.92 | 133 | 94 | 70 | 56 | 47 | 40 | 35 | 31 | 28 |
| 0.94 | 130 | 92 | 69 | 55 | 46 | 39 | 34 | 30 | 27 |
| 0.96 | 127 | 90 | 67 | 54 | 45 | 38 | 34 | 30 | 27 |
| 0.98 | 125 | 88 | 66 | 53 | 44 | 38 | 33 | 29 | 26 |
| 1.00 | 122 | 86 | 64 | 52 | 43 | 37 | 32 | 29 | 26* |

MCCD - medical certification of cause of death; ^after adjusting for the prevailing coverage of routine death surveillance (death registration along with MCDD); *updated at 08 00 hours; **Prevailing coverage in 2018 (most recent data)

**Suppl Table 4. Infection fatality ratio matrix using reported versus estimated COVID-19 deaths in the numerator, India, 31 July 2020**

| Correction factor to adjust for prevailing death surveillance and errors in MCCD | Scenarios of case detection ratio  (per 100 cases) | | | |
| --- | --- | --- | --- | --- |
|  | **1** | **2** | **5** | **10** |
| None* | 0.02 | 0.04 | 0.11 | 0.21 |
| 5.5^ | 0.12 | 0.23 | 0.58 | 1.16 |
| 8.3^ | 0.17 | 0.35 | 0.87 | 1.75 |
| 11^ | 0.23 | 0.46 | 1.16 | 2.32 |

*using reported COVID-19 deaths in the numerator; ^using estimated COVID-19 deaths in the numerator
